# Electrophysiological Changes Preceding the Onset of Atrial Fibrillation after Coronary Bypass Grafting Surgery

**DOI:** 10.1371/journal.pone.0107919

**Published:** 2014-09-23

**Authors:** Feng Xiong, Yalin Yin, Bruno Dubé, Pierre Pagé, Alain Vinet

**Affiliations:** 1 Research Center, Hôpital du Sacré-Cœur de Montréal, Université de Montréal, Montréal, Canada; 2 Montréal Heart Institute, Université de Montréal, Montréal, Canada; 3 Biomedical Engineering Institute, Université de Montréal, Montréal, Canada; 4 Department of Surgery, Université de Montréal, Montréal, Canada; Temple University, United States of America

## Abstract

**Background:**

The incidence of Post-CABG atrial fibrillation (AF) lies between 25% and 40%. It worsens morbidity and raises post-operative costs. Detection of incoming AF soon enough for prophylactic intervention would be helpful. The study is to investigate the electrophysiological changes preceding the onset of AF and their relationship to the preoperative risk.

**Methods and Results:**

Patients were recorded continuously for the first four days after coronary artery bypass grafting surgery (CABG) with three unipolar electrodes sutured to the atria (AEG). The patients experiencing an AF lasting more than 10 minutes were selected and the two hours before the onset were analyzed. Four variables were found to show significant changes in the two hours prior to the first prolonged AF: increasing rate of premature atrial activation, increasing incidence of short transient arrhythmias, acceleration of heart rate, and rise of low frequency content of heart rate. The main contrast was between the first and last hour before AF onset. Preoperative risk was not predictive of the onset time of AF and did not correlate with the amplitude of changes prior to AF.

**Conclusions:**

Post-CABG AF were preceded by electrophysiological changes occurring in the last hour before the onset of the arrhythmia, whereas none of these changes was found to occur in all AF patients. The risk was a weighted sum of factors related to the density of premature activations and the state of atrial substrate reflected by the sinus rhythm and its frequency content prior to AF. Preoperative risk score seems unhelpful in setting a detection threshold for the AF onset.

## Introduction

Coronary artery bypass graft surgery (CABG) is performed to relieve angina, bypass atherosclerotic narrowing and improve blood supply to coronary circulation [1]–[5]. Currently about 500,000 CABG operations are carried out each year in United States. The incidence of post-CABG atrial fibrillation (AF) has been reported to be in the range of 25% to 40%, most often occurring in the second or third post CABG surgery day [6]–[8]. Postoperative AF is associated with worse morbidity, as well as longer and more expensive intensive-care hospitalization [9]–[14]. In the United States, the cost for intensive care for postoperative AF is substantial, with estimated annual expenditures exceeding 1 billion dollars [13], [14]. Prediction of incoming AF after CABG soon enough to allow for prophylactic intervention would thus be helpful and cost-effective [2], [3], [12], [13].

The fundamental mechanisms responsible for AF, especially for post-surgery patients, is still not well understood [2], [3], [12]. Electrical properties, such as heterogeneous spatial distribution of excitability and repolarization, may play an important role in the generation and perpetuation of the arrhythmia [15]–[17]. Many cardiac pathological changes may occur following CABG surgery [18]. These can enhance the heterogeneous spatial distribution of excitability and repolarization, thereby facilitating the occurrence of AF [19]–[23]. Studies to identify pre-, peri-, and postoperative risk factors have led to different and even controversial results [24]–[28]. Part of these discrepancies might originate from patients choice. The present study considers patients who did not have an AF diagnosis prior to surgery. It is based on the analysis of continuous post-operative recording of atrial electrograms to identify electrophysiological changes that might precede the AF onset, complemented by investigation of preoperative risk factors.

## Material and Methods

### Study Group

Patients admitted for CABG surgery from 1999 to 2004 at Hôpital du Sacré-Coeur de Montréal (HSC) and Institut de Cardiologie de Montréal (ICM) were screened. The protocol was approved by the Ethics Committee of Hôpital du Sacré-Coeur de Montréal (CE-95-11-69). Written consent was obtained from all patients. To document the process, the consent forms were kept in separate research files. Exclusion criteria were: not in sinus rhythm at admission, taking class I or III antiarrhythmic drugs or digoxin, having a prior history of AF, having congestive heart failure, receiving hemodialysis, or having a permanent pacemaker. A total of 137 patients were selected, 108 from HSC and 29 from ICM. The pre- and peri-operative available data were: age, sex, left ventricular ejection fraction (LVEF, insufficient if LVEF<60%), diagnosis of hypertension (HT), diabetes, chronic obstructive pulmonary disease (COPD), history of stroke, prior myocardial infarct (MI), serum creatinine level, preoperative use of beta-blocker, calcium channel inhibitor or vasopressor/inotrope, number of vessels at CABG surgery, beating heart or extra-corporal circulation used during CABG, duration of extracorporeal circulation, duration of aortic clamp time. Patients who experienced an episode of AF lasting for more than 10 minutes in the first 4 post-operative days were classified as AF patients, and the others as Non-AF patients. 41 patients were classified as AF patients. Table 1 gives the distribution of the baseline characteristics in AF and Non-AF groups and the *p* values of the univariate logistic regression.

**Table 1:** Demographic and surgical data.

Group	AF	Non-AF	*p*
Number (n, %)	41 (29.93%)	96 (70.07%)	
Age (years, mean±std)	68.54±7.40	62.42±9.19	<0.001
Sex (n, % among men/women)			0.939
Men	31 (29.8%)	73 (70.2%)	
Women	10 (30.3%)	23 (69.7%)	
LVEF (n, %)	7 (17.07%)	11 (11.46%)	0.197
Stroke (n, %)	4 (9.75%)	4 (4.16%)	0.222
MI (n, %)	23 (56.09%)	39 (40.62%)	0.007
COPD (n, %)	4 (9.75%)	10 (10.42%)	0.827
Hypertension (n, %)	30 (73.17%)	56 (58.33%)	0.072
Serum Creatinine (mean±std, mmol/L)	101.08±38.170	89.29±28.793	0.031
Diabetes (n, %)	13 (31.71%)	31 (32.29%)	0.575
Mean Number of Vessels of CABG surgery (Mean)	2.63	2.63	0.727
Beating Heart vs. Extracorporeal Circulation (n, %)	4 (9.75%)	10 (10.42%)	0.820
Extracorporeal Circulation Duration (minutes, mean±Std,)	71.07±38.89	66.27±34.54	0.528
Cross-clamp Duration (minutes, mean±Std)	44.05±26.09	42.94±25.66	0.682
Preop. Treatment (n, %)			
Beta-Blockers	28 (68.29%)	77 (80.21%)	0.474
Calcium Channel Blockers	11 (26.83%)	23 (23.96%)	0.444
Vasopressor/Inotropes	2 (4.87%)	3 (3.13%)	0.640

LVEF: left ventricular ejection fraction;

MI: prior myocardial infarct;

COPD: chronic obstructive pulmonary disease;

n is the number of the specified type of patients;

% represents the percentage among the AF or Non-AF group if not specified.

### Preoperative Risk Factor

The distribution of the first sustained AF duration time was very inhomogeneous, ranging from 10 to 3732 minutes with a median of 353 minutes. Some of the Non-AF patients had short transient supraventricular arrhythmias, lasting from several seconds to 2 or 3 minutes with a maximum duration of 5 minutes. Association between preoperative data and AF was investigated by logistic regression. The effects of clinical variables and premature atrial activations on AF onset time was studied by Cox regression [29].

### Variables Extracted from AEG in AF and Non-AF Patients

The recording device was a modified (class III) three-channel Holter digital recorder (Burdick, model 6632). Three unipolar electrodes (ETHICON model TPW40) were sutured on the atria epicardium and connected to the positive poles of the Holter by wires fixed on the patient's thoracic wall. Three negative poles were connected together to serve as a reference electrode positioned on the lateral side of the thigh with an adhesive skin solid gel electrode. In the most common setup, two electrodes were sutured on the right atrium and one on the left atrium. The sampling rate was 500 Hz per channel.

The main goal of this paper is to analyze changes that may occur before the onset of post-operative AF and, as such, the analysis of changes were firstly restricted to the group of patients having experience a prolonged (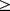
10 min) AF. To avoid the potential effects of anti-arrhythmic medications started after the AF onset, only the first sustained AF for each AF patient was considered and the two hours recordings before the onset were analyzed. 29 patients had analyzable three channels recordings and were selected for AEG analysis.

To verify the results obtained from AF patients, a control group of Non-AF patients was formed. Each AF patient was matched with two Non-AF patients, resulting in a group of 58 Non-AF patients. The priority order of matching criteria was preoperative risk score, date of surgery and gender. The criterion of risk score was intended to get the same distribution of preoperative risk in both groups. The criterion of date of surgery was aimed at bringing homogeneity in the surgery and post-surgery handling. The sex criterion was to reduce the differences that may be associated to gender. For Non-AF patients, the two hours corresponding to the same post-operative time than the matched AF patient were selected for analysis. Variables showing significant temporal changes among AF patients were analyzed using the matched 2 hours AEG recording of the Non-AF patients.

**Figure 1:**
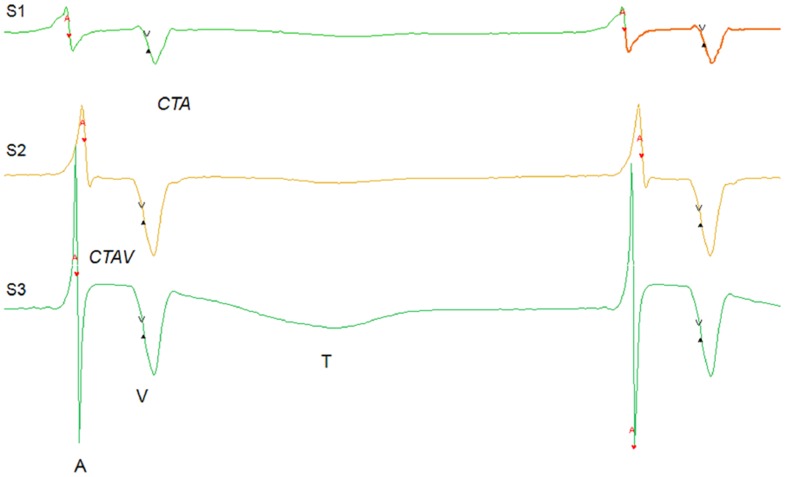
An example of recorded atrial electrogram (AEG): three channels (S1: superolateral right atrium. S2: inferior right atrium; S3: superior left atrium.) AEG in 2 consecutive normal sinus beats. The atrial (A) and ventricular (V) activations are indicated, as well as the ventricular T wave (T). *AA*, *CTA* and *CTAV* are also indicated.

AEG records both local atrial activation (A) and far field ventricular activation (V) (see Figure 1). Since atrial activations are produced by the travelling of activation front close to the electrodes, they are not simultaneously throughout the recording channels. The order in which the channels activate provides information on the origin of the activation that can be used to separate normal sinus from abnormal activations. The atrial period of activation (*AA*: difference between the times of the first activation in consecutive beats), the intra-atrial conduction time (*CTA*: time between the first and last A within a beat) and the atrio-ventricular conduction time (*CTAV*: time elapsed from the last A to the following V within a beat) were computed for each beat.

Times of atrial (A) and ventricular (V) electrical activations were detected by using a dedicated method [30]. Atrial and ventricular activations of different channels belonging to the same beat were grouped together, and classified as normal sinus beats, premature atrial activation (*PAA*), premature ventricular activation (*PVA*), and episodes of ventricular or atrial arrhythmia (see detailed definition below). The detection and classification were finally validated using in-house software.

*PAA* were defined by two criteria: either the atrial firing order was different from that of normal sinus beats, or *AA* was less than 70% of the mean *AA* of normal sinus beats in the preceding five minutes. Rate of *PAA* (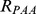) in a reference period (e.g. 5 minutes) was calculated as the number of *PAA* divided by the duration of the reference period, excluding intervals of atrial and ventricular arrhythmias. An episode of arrhythmia was considered to occur when there were more than 3 consecutive atrial or ventricular ectopic beats. In this case, the first ectopic beat was kept as a *PAA* or *PVA*, while the others were joined in an episode of arrhythmia.

The two-hour recordings were partitioned in 5 minutes intervals. All events different from normal sinus beats were excluded, as well as the first sinus beat immediately following a PAA or an episode of arrhythmia. The remaining normal sinus beats in each interval were used to calculate the mean AA (*AAMean*), its standard deviation (*AAstd*), the root mean square of differences between the successive AAs (*rMSSD*), the proportion of successive beats with a difference>50 ms (*pNN50*), mean intra-atrial (*CTAMean*) and atrio-ventricular conduction time (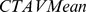), and correlation between *AA*, *CTA* and *CTAV* time series, referred as *CorrAA_AV*, *CorrAA_CTA*, and *CorrAV_CTA* respectively. The spectral analysis of the AA was used to study the cardiac autonomic nervous system [31]–[37]. For each interval of 5 minutes, *AA* time series of normal sinus beats were extracted and detrended with a cubic spline. The detrended series were then resampled by interpolation with a fixed time step of 0.25 second. Successive windows of 512 points were considered, with a 256 points overlap. Results of the windows within each five minutes interval were averaged. Each time series of 512 points was convoluted with a Hamming window. Yule-Walker auto regression method was used to compute the power spectrum of the resulting time series. Following common rules in heart rate variability (*HRV*) analysis [35], [37]–[39], power spectrum contents of the low (*LF*: 0.04–0.15 Hz) and high (*HF*: 0.15–0.40 Hz) frequencies components were calculated, as well as *LFPortion* (*LF*/(*LF*+*HF*)), *HFPortion* (*HF/(LF+HF)*), *LF/HF* and *HF/LF* Ratios. For some analysis, time partitions from 10 to 60 minutes were also considered. In these cases, the values assigned to each interval were the mean of 5 minutes intervals enclosed in each period.

An alternative method was developed to normalize data for each patient. For *AA*, *CTA* and *CTAV*, each data point was replaced by its position (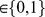) in the cumulative distribution of values obtained for each patient during the two hours, and then the mean position was calculated for each 5 minutes interval. For *PAA* rate, arrhythmia duration, *AA* standard deviation, *LF*, *HF*, *LF* Portion and *HF* Portion, *LF/HF* and *HF/LF* ratio, *CTA*, *CTAMean*, *CTAStd*, *CTAV*, *CTAVStd*, *CorrAA_AV*, *CorrAA_CTA*, *CorrAV_CTA*, the values were obtained for each five minutes interval, and position was allocated with respect to the set of 24 values obtained from the two hours. The rule to compute values for>5 minutes partitions was the same as for 5 minutes data. In the sequel, these are designated as position data.

Cluster analysis was also use to compare the time course of different variables among patients. Pearson's correlation coefficient was used as similarity measure. If a patient temporal pattern of change was significantly (p
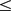
0.5) correlated with the mean profile of an existing cluster, it was added to the cluster [40].

## Results

### Preoperative Risk Factor Analysis

It might be conjectured that the nature and amplitude of the changes needed to trigger an AF episode depend on the level of pre- and peri-operative risk. The data described in Table 1, available for 137 patients, were used to produce a preoperative risk score via logistic regression.

#### Preoperative Risk Score by Multivariate Logistic Regression Analysis

The study group had more men than women (104 vs. 33), but the proportion of AF patients among men or women was similar (29.8% vs. 30.3%). Analyses were performed using univariate and forward conditional multivariate logistic regression. For multivariate analysis, the model entry and retention criteria were set at p<0.1 and p<0.15. The final logistic models were evaluated using the Hosmer-Lemeshow goodness-of-fit test [41]. Both unweighted and weighted versions were tried, the latter to alleviate the men vs. women disproportion by multiplying women contribution to the likelihood function by the men to women ratio. Since there were only minor differences between the two types of models (same choice of variables, slight changes in parameters values and significance), only the results of the weighted versions are reported.

**Figure 2:**
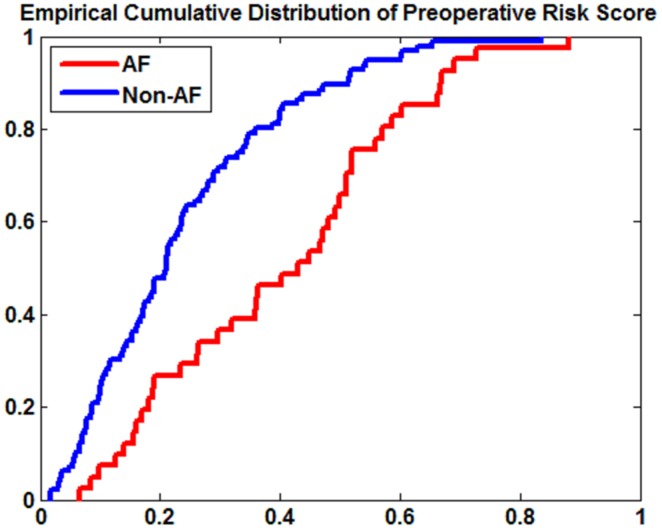
The empirical CDF (cumulative distribution function) of preoperative risk score of AF and Non-AF groups.

As seen in Table 2, four variables were identified as potential predictors by univariate logistic regression: age (p<0.001), prior myocardial infarct (p = 0.007), level of serum creatinine (p = 0.03), hypertension (p = 0.072). Three of these were kept in the multivariate model: 1) Age (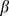 = 0.101; p<0.001); 2) Myocardial Infarct (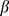 = 0.711; p = 0.036); 3) Serum Creatinine (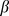 = 0.012; p = 0.025). Hypertension was not included because it was correlated with age. The sensitivity and specificity, calculated from the optimal threshold established from the ROC operating curve built with the risk score, were 60.98% and 79.17% respectively. The low sensitivity, especially in the men group, was a consequence of the wide distribution of the preoperative risk scores among AF patients (Figure 2).

**Table 2:** Beta values and significance of the variables in the logistic regression univariate and multivariate models.

		Univariate	Multivariate
Age	β	0.0847	0.101
	p	<0.001	<0.001
MI	β	0.8331	0.711
	p	0.0077	0.036
Serum Creatinine	β	0.0123	0.012
	p	0.0317	0.025
HT	β	0.6318	
	p	0.072	

MI(prior myocardial infarct);

HT (hypertension),

#### Cox Regression of Preoperative Risk Factor vs. Time of AF Occurrence

**Figure 3:**
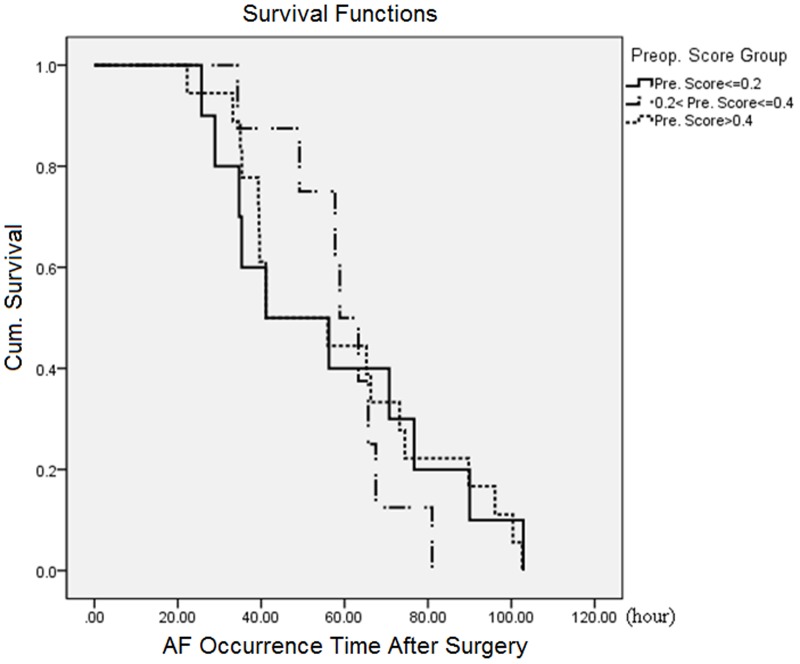
Survival curves as a function post-operative time of AF onset. The survival curves of three groups AF patients defined by their preoperative risk score: 1) 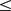
0.2; 2) 0.2<P
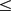
0.4; 3)>0.4.

Cox regression was used to investigate the effect of the preoperative score upon the AF onset time. Figure 3 shows the survival curves of AF patients for three groups defined by the preoperative risk score: 1) 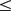
0.2 (11 AF patients); 2) 0.2<P
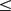
0.4 (8 AF patients); 3)>0.4 (22 AF patients). AF started to occur in the second day, with a strong incidence in the second and third day. The higher and lower risk groups evolved together, illustrating the absence of relation between the time of AF onset and the preoperative risk, which was confirmed by its non-significance in the Cox regression (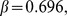 p = 0.45) and the weak, non-significant correlation between the two variables (r = −0.107, p = 0.54). This suggests that the preoperative variables can, to a certain extent, predict who will get AF, but not the time when AF will occur.

### Preoperative Risk Factor Analysis vs. Premature Atrial Activation (PAA)

**Figure 4:**
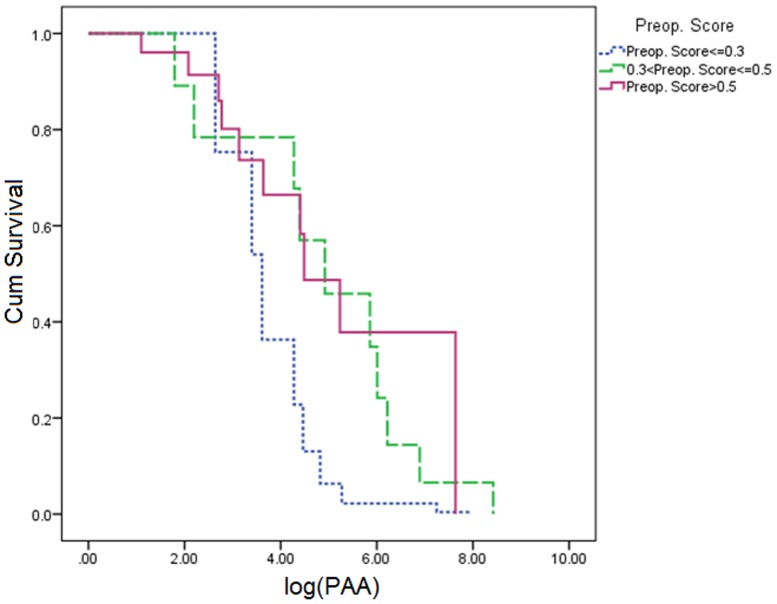
Survival curves as a function of the logarithm of the total number of PAA experienced before AF onset in 3 groups defined by their preoperative score 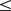
0.3, [0.3–0.5], and >0.5.

PAA are known to be associated to the occurrence of AF [42]–[45]. For the 29 patients with complete 2-hours available data, the AF episode was found to be triggered by a PAA, which most often originated from the left atrium (26/29 patients). However, the total number of PAA during the two hours varied widely among patients. It had a long-tail distribution with [*n_PAA_*_,min_., *n_PAA_*_,median_, *n_PAA_*_,mean_, *n_PAA_*_,max._]  =  [3,84,556,4539] and four patients with *n_PAA_* 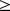 1400. It can be hypothesized that the higher risk patients might either be more prone to produce PAA or more vulnerable to PAA. Cox regression of the number of PAA experienced before AF vs. preoperative risk was not significant (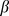 = 1.363, p = 0.173). Figure 4 shows the evolution of the cumulative number of PAA before AF in three groups with increasing preoperative risk: 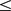
0.3, 9 patients, [0.3–0.5], 10 patients,>0.5, 10 patients). The three intertwined curves suggest that the preoperative risk score has no relationship with the number of PAA before onset of AF, as confirmed by Cox's regression.

### Time Evolving Analysis of Variables Extracted from AEG

The two-hours preceding AF onset were analyzed as described in the section of “Variables Extracted from AEG in AF and Non-AF Patients”. In addition to *PAA* rate and transient arrhythmia duration time, the variables related to the normal atrial activations were included: *AAMean*, *AAstd*, *rMSSD*, *pNN50*, *LF*, *HF*, *LFPortion*, *HFPortion*, *LF/HF, HF/LF*, *CTAMean*, *CTAStd*, *CTAVMean*, *CTAVStd*, *CorrAA_AV*, *CorrAA_CTA*, and *CorrAV_CTA*.

#### ANOVA and Post-hoc Analysis of Time Variables (Raw Data and Position Data) Before AF Onset

Time evolution of each variable was analyzed using repeated measure ANOVA [46]. Multivariate normality test of repeated measures was done for each variable using the Shapiro-Wilk test [47]. Non-normality was diagnosed except for *LFPortion* and *HFPortion*. Logarithm transformation of variables was performed to improve the normality distribution, except for *PAA* rate and arrhythmia duration which had some zero values. Upon repeated measures ANOVA, seven variables were diagnosed to have significant (p<0.05) time effects: *PAA* rate (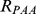), transient atrial arrhythmia duration (*ArrhyDuration*), mean of sinus atrial activation interval (*AAMean*), low frequency portion of AA time series (*LFPortion*), high frequency portion of AA time series (*HFPortion*), low to high frequency ratio (*LF/HF*), and high to low frequency ratio (*HF/LF*). The same variables were significant for the analysis of position data, but most often with higher significance level (Table 3). The pairs (*LFPortion, HFPortion*) and (*LF/HF, HF/LF)* are redundant, but may have slightly different p values upon logarithm transformation. Post–hoc analysis of orthogonal contrasts detected a significant difference between the first and the second hour for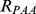, arrhythmia duration and *AAMean*, while only the contrasts involving the last 30 minutes were significant for *LFPortion*, *HFPortion*, *LF/HF*, *HF/LF*.

**Table 3:** ANOVA and contrast analysis of the raw and position data of variables with significant time-effects in 2-pre AF hours.

		Time	[120 60] vs. [60 0]	[60 30] vs. [30 0]	[30 15] vs. [15 0]	[10 5] vs. [5 0]
raw	*RPAA*	0.035	0.019↑			
	*ArrhyDuration*	0.03	0.016↑			0.01↑
	AAMean*	0.005	0.012↓	0.025↓	0.051↓	
	LFPortion*	0.005	0.004↑		0.035↑	0.032↑
	HFPortion*	0.005			0.002↓	0.001↓
	LF/HF*	0.028			0.003↑	0.001↑
	HF/LF*	0.028			0.003↓	0.001↓
Position	RPAA*	0.001	0.030↑			0.002↑
	ArrhyDuration*	0.001	0.034↑			0.002↑
	AAMean*	0.005	0.023↓	0.052↓	0.017↓	
	LFPortion*	0.043	0.049↑		0.026↑	0.005↑
	HFPortion*	0.018			0.008↓	<0.001↓
	LF/HF*	0.037			0.016↑	0.005↑
	HF/LF*	0.045		0.049↓	0.036↓	0.001↓

The upward and downward arrows stand respectively for the increasing and decreasing trend from one period to the next. “Time” column is the time effect significance in the two hours ANOVA analysis. The contrast analysis over the two time periods is listed subsequently. “0” is corresponding to the onset of AF.

Variables with asterisk were log transformed.

**Figure 5:**
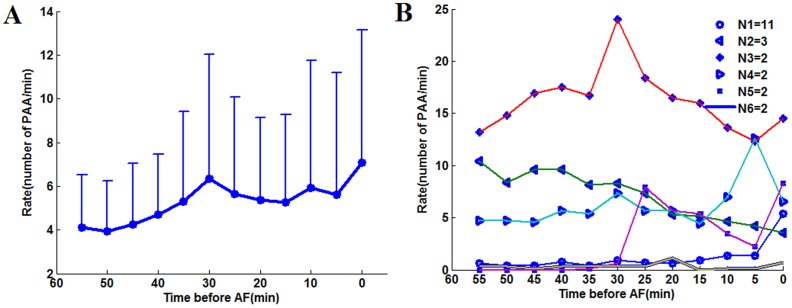
*R_PAA_* temporal evolution and the patterns of change. (A) Mean value and standard deviation of PAA rate within each 5 minutes in the last hour before AF; (B) Mean patterns associated to the clusters obtained by the analysis of the five minutes time series. The number of patients corresponding to each pattern is indicated in the legend. Only the patterns with more than one patient are shown. The abscissa is the time before the onset of AF (minutes).

**Figure 6:**
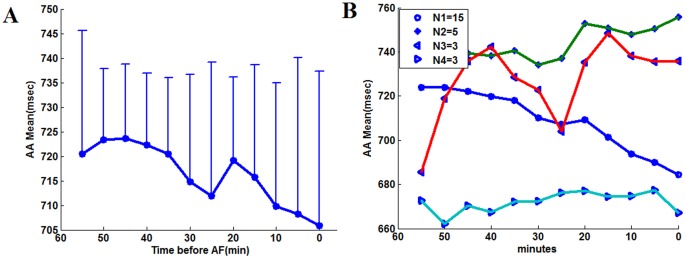
Temporal evolution and patter of changes of *AAMean* A: The trend of mean value of variables *AAMean*. B: Profiles obtained by cluster analysis.

Figure 5 shows the average *R_PAA_* temporal evolution (panel A), as well as the most common patterns of change during the last hour identified by cluster analysis (panel B). The last hour data was chosen because the contrast analysis showed that significant changes occurred in this time period. *R*_PAA_ mean profile suggested a continuous increase starting around 50 minutes before AF. However, the sustained increasing trend was not present in the cluster analysis results. The subgroup with the highest patient number rather demonstrated a modest increase in the last 30 minutes before AF. Similar analysis was repeated for *AAMean*. *AAMean* suggests a gradual and sustained acceleration of atrial rate, which was in fact presents in 15 patients (Figure 6). The phenomenon whereby the trend exhibited by the mean values can be found only in a subgroup patients was also found for the variables *ArrhyDuration*, *LFPortion*, *HFPortion*, *LF/HF* and *HF/LF.*

Sympathetic stimulation is known to decrease AA and might change *LFPortion* [48]–[53], which suggests that *AAMean* could be somewhat correlated to *LFPortion*, at least for patients with an accelerating or decelerating trend in sinus heart rate. However, the correlation coefficients between *AAMean* and *LFPortion* in the last hour before AF were widely distributed, ranging from −0.4 to 0.7. It was very low even for some of the patients with a marked decreasing AA time trend. It means that each variable can bring specific information since coordinated changes of all the variables were rare.

#### Analysis of Variables in Non-AF Control Patients

**Figure 7:**
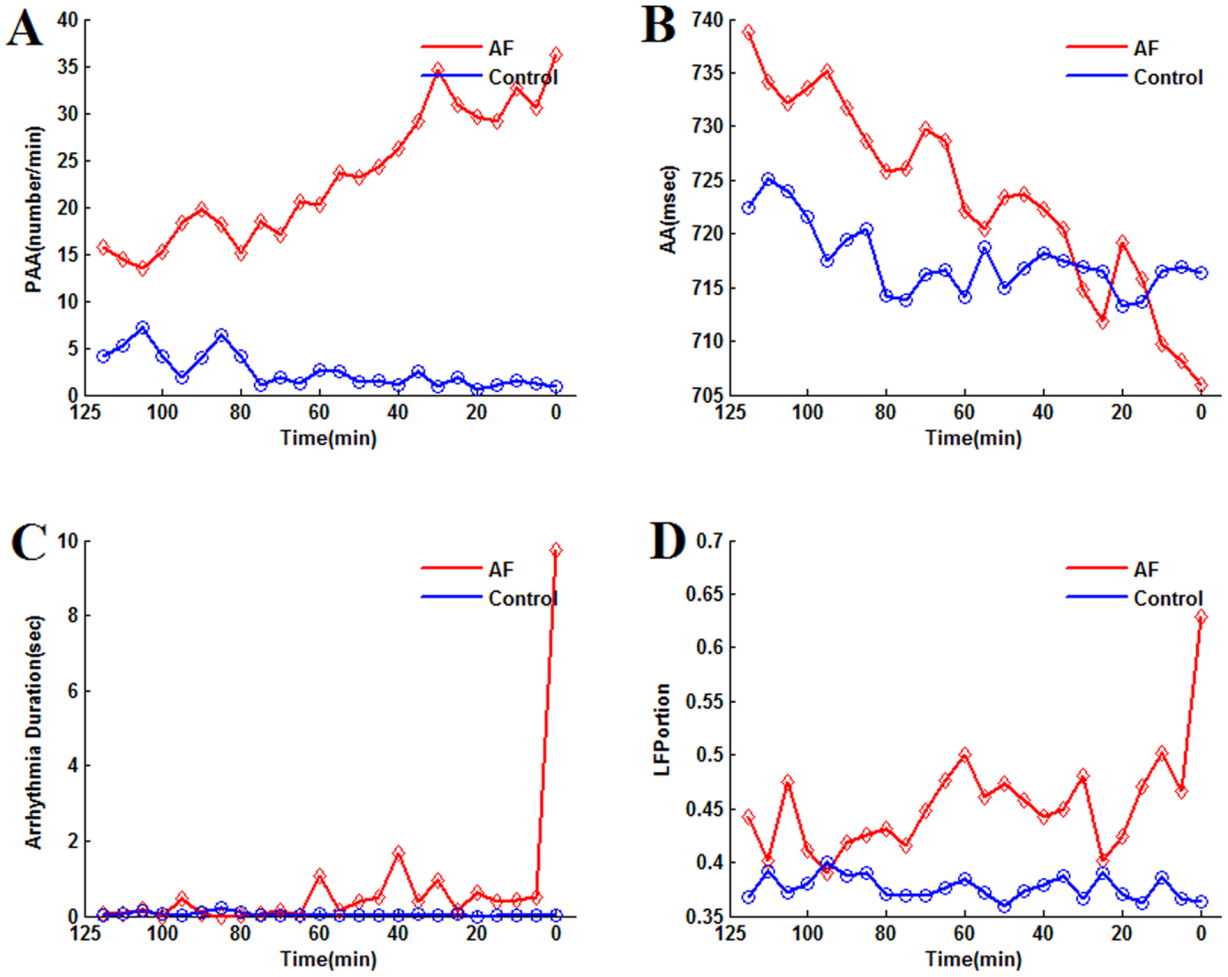
Mean value of *PAA* rate (A), *AA* (B), Arrhythmia duration (C), and *LFPortion* (D) within each 5 minutes for the 2 hours of Control (Non-AF) and AF groups.

The variables extracted from AEG showing significant temporal changes before AF onset were analyzed in Non-AF patients by the similar methods discussed above. Repeated measurement ANOVA analysis was applied to the raw data and logarithmic transformed data. In contrast to the AF group, there was no significant time effect in the two hour time series of the variables as *PAA* rate, *ArrhyDuration*, *AAMean*, *LFPortion*, *HFPortion*, *LF*/*HF*, *and HF*/*LF* (Table 4). As aforementioned, the AF group post-hoc analysis showed that significant contrasts existed among one or more periods like [120 60] vs. [60 0], [60 30] vs. [30 0], [30 15] vs. [15 0], or [10 5] vs. [5 0]. None of them was found to be significant in the Non-AF control group. Figure 7 illustrates the time evolution of *PAA*, *AA*, *ArrhyDuration* and *LFPortion*, which did not show any obvious increasing or decreasing trend in the Non-AF group.

**Table 4:** ANOVA analysis of time effect of the Non-AF patient variables data, which were significant in 2-pre AF hours in AF patient group.

		Original Data	Logarithmic Data
raw	*RPAA*	0.194	N/A
	*ArrhyDuration*	0.283	N/A
	*AAMean*	0.403	0.541
	*LFPortion*	0.364	0.354
	*HFPortion*	0.364	0.438
	*LF/HF*	0.438	0.360
	*HF/LF*	0.438	0.360
Position	*RPAA*	0.329	0.402
	*ArrhyDuration*	0.235	0.267
	*AAMean*	0.330	0.866
	*LFPortion*	0.299	0.067
	*HFPortion*	0.299	0.407
	*LF/HF*	0.299	0.067
	*HF/LF*	0.299	0.407

N/A: not applicable.

The same variables were also analyzed by two-way ANOVA (within: Time, between: AF vs. Non-AF group, Table 5). As expected for the lack of time effect in the Non-AF group, there was significant (or close to significance for *AAMean*) group*time interactions when either the two hours or the last hour before AF were considered. However, all variables, except *AAmean*, also showed a significant group effect. This group effect was also present in the first hour, except for *ArrhyDuration*. Since the group effect compares the mean values of all time intervals, it was more meaningful to the first hour period in which time effect was absent for most variables.

**Table 5:** Two-way ANOVA analysis of time effect, and group effect between AF and Non-AF Patients.

		Group (AF vs. Non-AF)			Time			Group*Time		
		2 hrs.	1^st^ hr	2^nd^ hr	2 hrs.	1^st^ hr	2^nd^ hr	2 hrs.	1^st^ hr	2^nd^ hr
Original Data	*RPAA*	0.000	0.009	0.000	0.008	0.710	0.042	0.000	0.160	0.021
	*ArrhyDuration*	<0.001	0.329	0.000	0.478	0.915	0.437	<0.001	0.329	<0.001
	*AAMean*	0.847	0.702	0.998	0.001	0.045	0.070	0.072	0.773	0.080
	*LFPortion*	0.001	0.034	<0.001	<0.001	0.129	<0.001	<0.001	0.007	<0.001
	*HFPortion*	0.001	0.034	<0.001	<0.001	0.129	<0.001	<0.001	0.007	<0.001
	*LF/HF*	<0.001	<0.001	<0.001	<0.001	0.011	<0.001	<0.001	<0.001	<0.001
	*HF/LF*	0.272	0.058	0.754	0.124	0.491	0.118	0.030	0.275	0.062
Logarithmic Transform	*AAMean*	0.747	0.619	0.887	0.136	0.066	0.538	0.289	0.525	0.090
	*LFPortion*	0.144	0.717	0.011	0.018	0.085	0.004	<0.001	0.001	<0.001
	*HFPortion*	<0.001	0.002	<0.001	<0.001	0.027	<0.001	<0.001	<0.001	<0.001
	*LF/HF*	0.009	0.154	<0.001	<0.001	0.264	<0.001	<0.001	0.025	<0.001
	*HF/LF*	0.009	0.154	<0.001	<0.001	0.264	<0.001	<0.001	0.025	<0.001

*RPAA*, *ArrhyDuration* are not applicable to logarithmic transformation.

Taken together, the results of the one-way and two-way ANOVA can be summarized as follows:

- in the AF group, *PAA* rate, *ArrhyDuration* and the low frequency content of *AA* variation tended to increase between the first and the second hour, while remaining constant in Non-AF patients;
- Except for *ArrhyDuration*, mean *PAA* rate and the low frequency content indices were higher in the AF group in the first hour. This suggests a higher level of dysfunctional autonomic neural balance and of atrial ectopic beats for AF patients that are further enhanced before AF onset. *AAMean* was shown to decrease before AF in about half of the patients (Figure 6). Figure 7B may suggest that *AAMean* was lower of Non-AF group in the first hour, but the high variance made this difference not statistically significant for raw or log-transform data.

#### Discrimination of Trigger and Non-Trigger Period

In an alternative approach, the capacity of variables to discriminate the closest time period to AF onset (triggering period) from others (non-triggering periods) was assessed by forward conditional stepwise logistic regression. The analysis was repeated for both raw and position data dividing the 2 pre-AF hours in equal time periods of 5, 10, 15, 20, 30 and 60 minutes.

**Figure 8:**
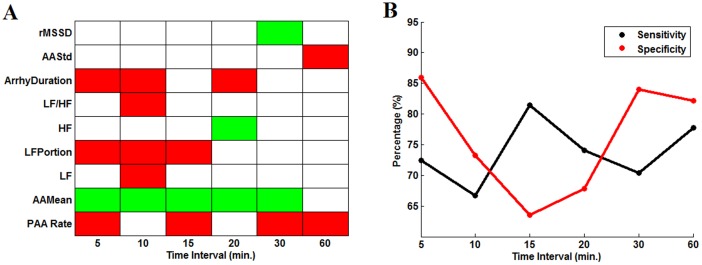
Results of logistic regression to discriminate trigger and non-trigger period. A) Results of the forward stepwise logistic regression using position data. Sign of estimated coefficient of predictors in the logistic model (red: positive coefficient, green: negative coefficient) for different partitions of 2 hours intervals before AF. B) Sensitivity (red, % of correct classification of the last interval) and specificity (black) of each model with cutting point calculated from the ROC curve.

Scores of the logistic model were used to build ROC curves and select the optimal cut-off point. The discrimination was much better using position data than raw data, even though the same variables were selected. The results obtained by position data are shown in Figure 8. Panel A gives the variables kept in the model for each time partition, the color code indicating whether higher (red) or lower values (green) were predictors of the triggering period. Four variables as 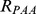, 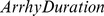, 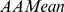 and *LFPortion* were more often present and should bring independent information. These were among the variables identified by Anova to have a significant time effect. The presence of 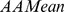 and *LFPortion* confirms, as aforementioned, that they bring independent information. The sensitivity and specificity (correct classification of the trigger and non-trigger intervals respectively) remained between 65% and 85% for all time partitions. Globally, the results suggested that AF incidence tends to be preceded by an increased number of PAA and transient arrhythmia episodes, on a background of accelerated sinus rhythm and a relative increase of its low frequency fluctuations. However, these changes did not occur simultaneously in all patients.

**Figure 9:**
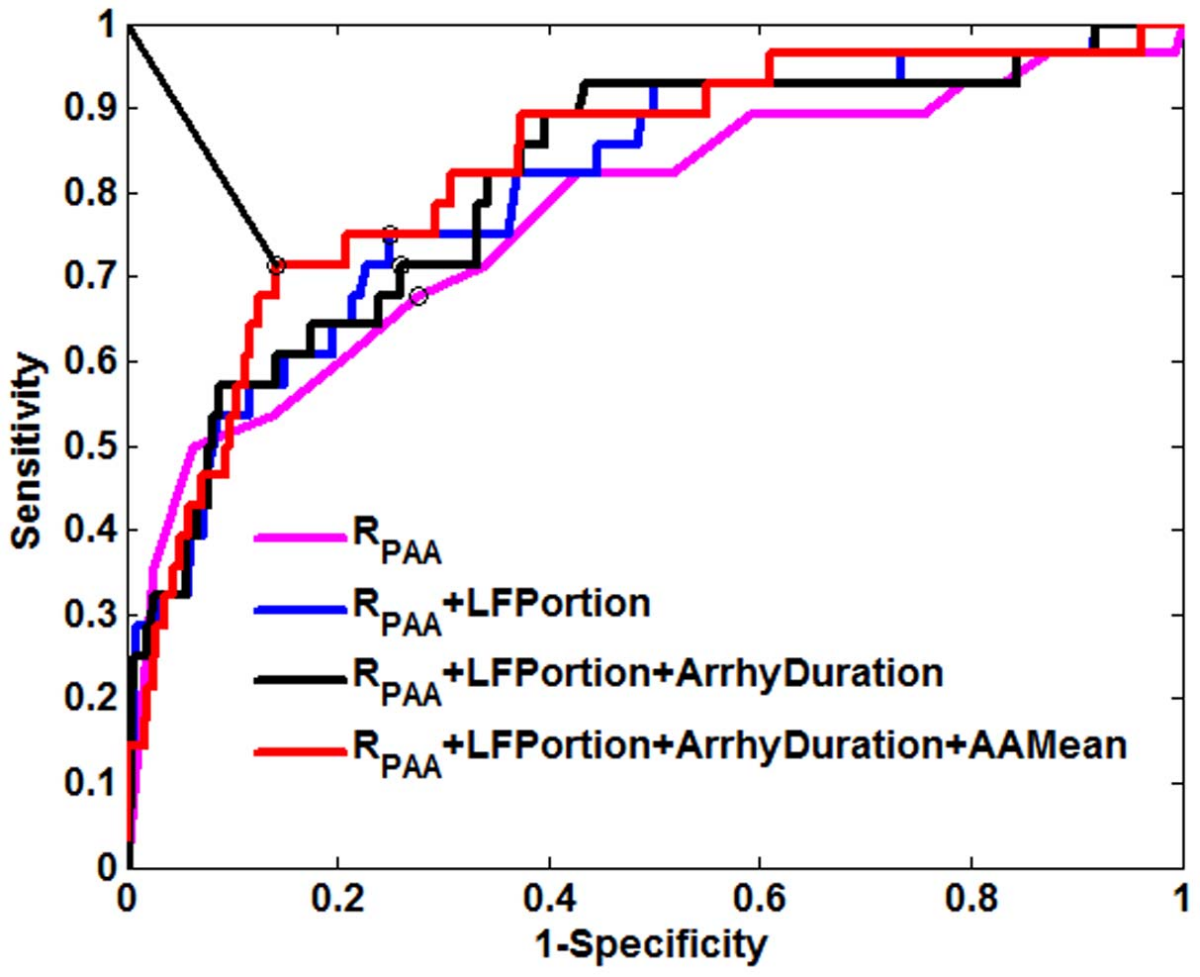
ROC curves of the scores obtained by stepwise logistic regression. ROC curves for the logistic regression models with successive inclusion of the variables: *RPAA, LFPortion, Arrhythmia Duration, AAMean*. The circles indicated the best cut off points for each model.

For 5 minutes interval partitioned data, the four variables were entered in the multivariate model in the following order*: RPAA, LFPortion, Arrhythmia Duration, AAMean*. Details of the univariate analysis are given in Table S1. Figure 9 shows the ROC curves associated with the successive models: I,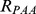; II, *LFPortion*+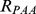; III, 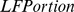 +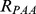
+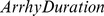; IV, 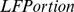
+ 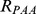
+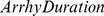
+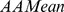. It is evident that the predictor 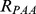 plays the most important role, achieving around 65% sensitivity and specificity. Then the other two predictors 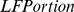 and 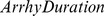 made some sensitivity improvements. The predictor of *AAMean*, finally introduced in the model, further improved the prediction, to reach a sensitivity of 71% and a specificity of 86%.

### Preoperative Risk Score vs. Postoperative Evolving Risk

It was assumed that patients with a higher preoperative score need less change to develop AF. Correlation analysis was done to check the relation between the preoperative risk score and the score of the last interval, the mean score of all time intervals, the difference between the score of the last interval and the mean score of all other intervals (Table S2). None of them was found even close to be significant. The preoperative score did not seem to be related to the dynamic changes preceding the onset of AF.

## Discussion

The mechanisms of the incidence of post-CABG AF are complex and still controversial. The present project is based on the analysis of multichannel epicardial electrograms to investigate electrophysiological changes preceding AF onset and their relationship to the preoperative risk factors. The main findings of the study are: The post-CABG AF was preceded by electrophysiological changes in the last hour before the onset of arrhythmia. The causes appear to be multifactorial, mainly involving increasing rate of premature atrial activation, short transient arrhythmias, accelerating heart rate, and rising low frequency content of heart rate. None of the above changes was found to occur in all AF patients.

### Preoperative Risk Factors

Diverse post-CABG AF risk factors have been identified, but results among different studies were often inconsistent, sometimes even controversial [54] [55] [56] [10], [18], [25], [57]–[61]. Beyond the fact that the mechanisms of AF occurrence are complex and multi-factorial, this might result from the fact that most studies were observational and retrospective, with different inclusion criteria.

For our study population, age appeared as the most important pre-operative predictor, the mean age of AF patients being around 6 years older than Non-AF patients (Men: 67 vs. 61 years, women: 75 vs. 68 years old). Similar results were reported by several other studies [55], [62]–[66]. The association could be attributable to age-related structural changes in the atrium such as dilation and fibrosis. These structural changes could influence the electrophysiological properties of atrial myocardium, such as prolonging atrial conduction times, increasing atrial stiffening, and splitting of atrial excitation wave in the pectinated trabecula [67], [68]. It has also been suggested that surgical trauma to sympathovagal fibers originating from the deep or superficial cardiac plexus during surgery may enhance age related pro-AF effect [62].

There are controversial reports about the effect of sex on postoperative AF [59], [69], [70]. There were around 3 times more men than women in our sample (104 vs. 33), but the incidence of AF was almost the same in the two groups. This explains why sex did not show up as a predictor in the current study. Women were on average older, but the age difference between AF and Non-AF patients was the same in the male and female groups. Sex did not appear in the multivariate logistic regression, even when an extra sex*age variable was added. This concurs with the conclusion of Auer et al. who indicated that sex was not an independent risk factor, but contradicts Zaman et al. who reached the inverse conclusion [55], [59], [71].

Hypertension is associated with left ventricular hypertrophy, which may impair ventricular filling, induce left atrial enlargement, slow down atrial conduction velocity, and increase cardiac tissue fibrosis and dispersion of atrial refractoriness. All these structural and electrophysiological changes predispose to AF [72]–[74]. Hypertensive patients in the current study had indeed a 78% increased risk of AF, an effect close to significance in univariate analysis. However, it did not appear as a predictor in the multivariate logistic model because the effect of hypertension was largely confounded by age, which contradicts the conclusion of Svedjeholm et al. [66].

Renal function impairment often induces an elevation of serum creatinine level [54]. The link between preoperative renal function and postoperative AF has been investigated in some studies. Patients with higher level of serum creatinine were more prone to postoperative AF [75], [76]. In our sample, this was found in the subgroup older than 60 years, whereas the relation was even reversed for younger patients. The result is difficult to explain, and may reflect the limited size of our study population.

Following MI, the injured heart tissue conducts electrical impulses more slowly, which can promote reentry following a PVC and retrograde conduction to the atria that may trigger AF [77]. In our sample, AF was not found to be triggered by PVC. Inversely, AF can often complicate MI acute myocardial infarction by reducing heart pump function [78]. In our study population, the age confounding effect was to some extent present for prior myocardial infarct (MI), but MI was still a predictor in the final multivariate model.

### Time Evolving Risk Factors

Different studies have investigated the dynamics of cardiac electrical recordings in the last two hours, one hour, thirty minutes, or even several minutes before the onset of post-CABG AF [79]–[81]. Some variables were found to have significant differences between the first and second hour before AF, or within the second hour, but never during the first hour. This suggests that the two hours before AF onset provided an appropriate time frame to analyze the evolving changes.

Globally, there was an *R_PAA_* increase in the last hour before AF onset. This is in agreement with the observations that atrial ectopic beats tend to be more frequent before the start of paroxysmal or postoperative AF [82]–[84]. However, analysis of the temporal *R_PAA_* evolution showed a sustained increasing trend was present only in a subgroup, which indicates that increasing PAA rate is not an absolute prerequisite to AF occurrence. Most patients also experienced an increased number of transient arrhythmia episodes. In contrast to *R_PAA_*, this was mainly restricted to the last 10 or 5 minutes before AF. In general, the arrhythmias were short and last less than 1 minute, such that longer duration of arrhythmia in a time period was most often resulting from the presence of multiple bursts. Even though arrhythmia durations were correlated with *R_PAA_* in the final period, both of them were predictors to discriminate trigger from non-trigger time periods.

The gradually accelerated heart rate during the last pre-AF hour coincides with previous observations that faster heart rate postoperative patients were at higher risk to develop AF [48], [85]–[87]. However, cluster analysis (Figure 6 B) revealed that this accelerating trend was only present in a subgroup of AF patients, the others having either a decreasing trend or constant heart rate. We would rather conclude, as Hogue et al., that there is no unique pattern wholly predictive of impending AF [88].

The LF and HF power spectral components of the heart rate variability are often considered to be a marker of sympathetic and/or parasympathetic modulation, although this point of view remains somewhat disputable [39]. Heterogeneous electrophysiological properties could be due to autonomic innervations [88], while their possible implication in postoperative AF is complex, sometimes even controversial [13], [48], [64], [86], [89]. It was reported that either vagal or sympathetic nerve stimulation, can decrease the atrial refractory period in a spatially heterogeneous way, thereby facilitating the occurrence of AF [90]–[94]. The difference between vagal and sympathetic stimulations might lie in the more spatially heterogeneous effect of vagal nerve activation. It has been put forward that elevated norepinephrine levels suggest sympathetic activation, while some other studies rather suggested divergent autonomic conditions to occur before arrhythmia onset, either heightened sympathetic or parasympathetic tone, or even dysfunctional autonomic heart rate control [13], [48], [64], [86], [89]. In both raw data and position data, we found an increasing trend of mean *LFPortion* in the last 30 minutes, reaching a peak in the last 10 minutes before AF. The increase of *LFPortion* might be ascribed to an augmentation of the sympathetic and/or decrease of the parasympathetic tone. However, the weak correlations between *AAMean* and *LFPortion* trend indicated that the evolving changes of these variables were not generally coordinated.

Position data always had higher level of statistical significance and classification accuracy. This comes from the huge variations of mean level and amplitude of changes observed for every variable among the patients. The normalization, based on the distribution of the values within each patient, removes the scale difference. It suggests that a relative threshold, adapted to the state of the patient, can be a better predictor of impending AF.

### Preoperative Risk Factors vs. Time Delay, PAA and Time Evolving Risk Scores

It might be hypothesized that AF patients with higher preoperative risks should develop AF sooner and with less PAA. It might also be conjectured that the time evolving score could be higher from the beginning or need less change to trigger AF. None of these hypotheses were supported by the data analysis. The preoperative score appeared to be a static risk measure, relatively good to classify Non-AF patients (around 80%), but not for AF patients (around 60%). The preoperative risk score was not found to be significantly related to the dynamic electrophysiological changes prior to AF.

### Study Limitation

The results of our study can only be considered as indicative because of the relatively small sample size owing to the limited data availability. The performance of both the preoperative and time evolving model should be evaluated with independent and larger population of patients.

Accordingly, the future study needs to use fewer criteria to exclude patients during the screening process, so that the predicting method could be improved to be useful for a larger set of postoperative patients. Other variables might also be considered in next step, such as activation waveform, whose change has been reported to precede the onset of AF [19], [80]. Moreover, the monitoring of blood pressure could be useful, particularly in association with heart rhythm whose fluctuations could be a response to vascular events that must certainly occur after an open-heart surgery.

## Conclusion

Our results show that post-CABG AF is preceded by epicardial electrocardiographic changes occurring in the last hour before the onset of arrhythmia, which is a prerequisite for monitoring and provide enough time for prophylactic intervention. Giving that none of these changes was found to occur in all AF patients, the predictive score should be a weighted sum of factors related to the potential triggers of AF, such as PAA, to the state of the tissue in which they occur, as well as the heart rate and the frequency content of its fluctuation. The better performance of position data suggests that detection threshold must be adapted to the state of each patient. Measures of preoperative risk factors do not seem to be helpful in setting threshold. The relative invariance of the data during the first hour before AF suggest that data could be normalized using the distribution of values collected during an initial reference period. Ideally, the reference period could be at the end of the first day since the incidence of AF appears to be very low in this period and the patients parameters appear to be relatively stable. However, it remains to be verified whether and when the relative stability of the indices can be reached.

## Supporting Information

Table S1 **p-value from univariate and multivariate logistic regression model with position data and 5 minutes partition.**
(DOCX)

Table S2 **Relation of the preoperative risk with the evolutionary scores.** Pearson correlation of the preoperative risk, with the score of last interval (I), the mean score of all intervals (II), and the difference the score of the last interval and the mean of other intervals (III) for time partitions of 5, 10, 20, 30, 60 minutes. The values in parentheses are the two tailed significance values.
(DOCX)
