# Supplementary material for: Electrophysiological Changes Preceding the Onset of Atrial Fibrillation after Coronary Bypass Grafting Surgery
Source: PLoS One. 2014 Sep 23;9(9):e107919. doi: 10.1371/journal.pone.0107919 (PMC4172567; doi:10.1371/journal.pone.0107919)
Supplement: Table S1 — p-value from univariate and multivariate logistic regression model with position data and 5 minutes partition. (DOCX) [file pone.0107919.s001.docx]

Table S1 p-value from univariate and multivariate logistic regression model with position data and 5 minutes partition

|  | Univariate (p Value) | Multivariate (p<0.05) |
| --- | --- | --- |
| *RPAA* | <0.001 | 0.005 |
| *ArrhyDuration* | <0.001 | 0.047 |
| *AAMean* | 0.002 | 0.012 |
| *AAStd* | 0.576 |  |
| *rMSSD* | 0.81 |  |
| *pNN50* | 0.794 |  |
| *LF* | <0.001 |  |
| *HF* | 0.058 |  |
| *LFPortion* | <0.001 | 0.001 |
| *HFPortion* | <0.001 |  |
| *LF/HF* | <0.001 |  |
| *HF/LF* | <0.001 |  |
| *CTAVMean* | 0.631 |  |
| *CTAVStd* | 0.489 |  |
| *CTAMean* | 0.632 |  |
| *CTAStd* | 0.558 |  |
| *CorrAA_AV* | 0.279 |  |
| *CorrAA_CTA* | 0.316 |  |
| *CorrAV_CTA* | 0.732 |  |
