# Supplementary material for: Electrophysiological Changes Preceding the Onset of Atrial Fibrillation after Coronary Bypass Grafting Surgery
Source: PLoS One. 2014 Sep 23;9(9):e107919. doi: 10.1371/journal.pone.0107919 (PMC4172567; doi:10.1371/journal.pone.0107919)
Supplement: Table S2 — Relation of the preoperative risk with the evolutionary scores. Pearson correlation of the preoperative risk, with the score of last interval (I), the mean score of all intervals (II), and the difference the score of the last interval and the mean of other intervals (III) for time partitions of 5, 10, 20, 30, 60 minutes. The values in parentheses are the two tailed significance values. (DOCX) [file pone.0107919.s002.docx]

Table S2 Relation of the preoperative risk with the evolutionary scores. Pearson correlation of the preoperative risk, with the score of last interval (I), the mean score of all intervals (II), and the difference the score of the last interval and the mean of other intervals (III) for time partitions of 5, 10, 20, 30, 60 minutes. The values in parentheses are the two tailed significance values.

|  | I | II | III |
| --- | --- | --- | --- |
| 5 min. | 0.116 (0.550) | -0.028 (0.883) | 0.201 (0.346) |
| 10 min. | -0.015 (0.940) | -0.088 (0.649) | 0.132 (0.675) |
| 15 min. | 0.069 (0.731) | 0.151 (0.443) | 0.143 (0.759) |
| 20 min. | -0.008 (0.969) | 0.102 (0.606) | -0.018 (0.498) |
| 30 min. | -0.044 (0.826) | -0.051 (0.642) | -0.141 (0.529) |
| 60 min. | -0.150 (0.438) | -0.316 (0.312) | -0.108 (0.361) |
